# A model for national assessment of barriers for implementing digital technology interventions to improve hypertension management in the public health care system in India

**DOI:** 10.1186/s12913-021-06999-9

**Published:** 2021-10-15

**Authors:** Shivani A. Patel, Kushagra Vashist, Prashant Jarhyan, Hanspria Sharma, Priti Gupta, Devraj Jindal, Nikhil Srinivasapura Venkateshmurthy, Lisa Pfadenhauer, Sailesh Mohan, Nikhil Tandon

**Affiliations:** 1 Department of Global Health and Epidemiology, Emory University, Atlanta, USA; 2 Centre for Chronic Disease Control, New Delhi, India; 3 Public Health Foundation of India, Gurgaon, India; 4 All India Institute of Medical Sciences, New Delhi, India

**Keywords:** Hypertension, Management, Infrastructure, Digital technology, Public healthcare system, India

## Abstract

**Background:**

There is substantial interest in leveraging digital health technology to support hypertension management in low- and middle-income countries such as India. The potential for healthcare infrastructure and broader context to support such initiatives in India has not been examined. We evaluated existing healthcare infrastructure to support digital health interventions and examined epidemiologic, socioeconomic, and geographical contextual correlates of healthcare infrastructure in 544 districts covering 29 states and union territories across India.

**Methods:**

The study was a cross-sectional analysis of India’s Fourth District Level Household and Facility Survey (DLHS-4; 2012–2014), the most up-to-date nationally representative district-level healthcare infrastructure data. Facilities were the unit of analysis, and analyses accounted for clustering within states. The main outcome was healthcare system infrastructural context to implement hypertension management programs. Domains included diagnostics (functional BP instrument), medications (anti-hypertensive medication in stock), essential clinical staff (e.g., staff nurse, medical officer, pharmacist), and IT specific infrastructure (regular power supply, internet connection, computer availability). Descriptive analysis was conducted for infrastructure indicators based on the Indian Public Health Standards, and logistic regression was conducted to estimate the association between epidemiologic and geographical context (exposures) and the composite measure of healthcare system.

**Results:**

Data from 32,215 government facilities were analyzed. Among lowest-tier subcenters, 30% had some IT infrastructure, while at the highest-tier district hospitals, 92% possessed IT infrastructure. At mid-tier primary health centres and community health centres, IT infrastructure availability was 28 and 51%, respectively. For all but sub-centres, the availability of essential staff was lower than the availability of IT infrastructure. For all but district hospitals, higher levels of blood pressure, body mass index, and urban residents were correlated with more favorable infrastructure. By region, districts in Western India tended towards having the best prepared health facilities.

**Conclusions:**

IT infrastructure to support digital health interventions is more frequently lacking at lower and mid-tier healthcare facilities compared with apex facilities in India. Gaps were generally larger for staffing than physical infrastructure, suggesting that beyond IT infrastructure, shortages in essential staff impose significant constraints to the adoption of digital health interventions. These data provide early benchmarks for state- and district-level planning.

**Supplementary Information:**

The online version contains supplementary material available at 10.1186/s12913-021-06999-9.

## Background

High blood pressure is responsible for over 6.08 million deaths across low- and middle-income countries (LMICs) annually [1]. Timely diagnosis [2] and appropriate medical management [3] of high blood pressure are foundational evidence-based interventions to mitigate resulting poor health outcomes [2]. Digital technologies are being increasingly leveraged as a potential implementation strategy to widely deliver these evidence-based interventions at scale [4]. Examples of technologies include combinations of mobile health (mHealth) applications to facilitate community screening for blood pressure [5], SMS messaging to communicate with patients [6], usage of electronic medical records to track and manage patients requiring chronic care [7], and use of electronic decision support systems to assure adoption of guidelines-based care [8]. To achieve sustainable impacts on population health outcomes, the World Health Organization has called on member states to explore “how digital technologies could be integrated into existing health systems infrastructures and regulation, to reinforce national and global health priorities.” [9]

In India, hypertension prevalence is estimated to be 18% among adults aged 15–49 years [10], with estimates as high as 30% in some populations [11]. Hypertension is the third leading cause of death and disability combined in India and causes over 1.5 million deaths annually [11, 12]. Furthermore, a large fraction of individuals with hypertension remain unaware of their condition [10]. Hypertension is therefore prioritized by the Government of India in its non-communicable diseases (NCDs) community screening and management programming and comprehensive programming being implemented through Health and Wellness Centres [13, 14].

Hypertension screening and management must be embedded within the existing healthcare system to feasibly and effectively reach the Indian population. The public healthcare system in India is organized as a hierarchy of four tiers of facilities that include: sub-centres at the village-level (SC; lowest levels of skilled personnel and resources; tasked with blood pressure screening), primary health centres that serve several villages (PHCs; contain a physician; tasked with blood pressure diagnosis and providing basic medical treatment), community health centres that serve the administrative unit known as a block (CHCs; include NCD clinics and are central to the integration of NCD care into primary care), and district hospitals that serve an entire district (DH; include higher levels of trained personnel, specialists, and more sophisticated infrastructure). This model was designed to broaden coverage of health care within existing resources and with the aim of progressive referral from lower to higher levels of health care depending on the need of the individual patient and the availability of system resources (skilled human resources, infrastructure and services).

Within this system, the national government has invested in developing technological platforms to improve care for hypertension through its community-based NCDs Prevention, Screening, Control and Management Initiative under India’s Comprehensive Primary Health Care Program [15, 16]. Such government-sector initiatives are particularly important in rural settings, which have experienced steady increases in cardiovascular disease and other chronic comorbidities associated with high blood pressure [17] but whose healthcare infrastructure has historically focused on delivering care for maternal and child health as well as infectious disease control.

As with any complex intervention, digital technology strategies must be adapted for the local context and contextual modifications may be needed in order achieve similar digital technology effects as in previous contexts [18, 19]. At a minimum, population-level management of high blood pressure with or without technology requires a combination of the essential drugs and adequate healthcare personnel for appropriate administration of drugs [20]. Operationally, the responsibility to deliver healthcare and maintain healthcare infrastructure has been vested with districts [20], administrative units within states. Contextual characteristics of healthcare facilities and the broader community at a district level therefore may critically inform strengths and weaknesses in the current healthcare system as the nation moves towards scale up of hypertension care through technology-assisted approaches.

Several implementation science frameworks exist to describe and evaluate dimensions of context, variously defined [21–23]. We employ the Context and Implementation of Complex Interventions (CICI) framework [23] to evaluate the current healthcare infrastructure context to support digital health interventions for high blood pressure diagnosis and management in India. We further examine dimensions of epidemiologic, socioeconomic, and geographical context to assess the broader contextual correlates of health care infrastructure to support digital health technologies.

## Methods

### Data sources

The fourth round of the District Level Household and Facility Survey (DLHS-4) is the most comprehensive and latest nationwide health facility assessment conducted by the government of India with coverage across 26 states and 3 union territories with detailed district-level data. The survey was conducted between 2012 and 2014. Facility data were collected through four separate modules (including questionnaire, physical inspection, and assessing registers) designed for each facility type. Facility data were available for 1540 District and Sub-District Hospitals (DH), 4810 Community Health Centers (CHCs), 8540 Primary Health Centers (PHCs), and 18,367 Sub Centers (SCs). In 377 districts, we analyzed DH facilities meeting the Indian Public Health Standards (IPHS) minimum bed criterion of 101 beds; in 171 districts where no facility met this criterion, we analyzed sub-divisional/sub-district hospitals (minimum of 31 beds). Data from two states (Gujarat and Jammu and Kashmir) and four union territories (Dadra and Nagar Haveli, Daman and Diu, Delhi and Lakshadweep) were not available.

To characterize broader community context at the district-level, we combined data from the household survey of the DLHS-4, the Annual Health Survey (AHS) [24–26], and the fourth round of the National Family Health Survey (NFHS-4) [27]. The DLHS-4 household survey was conducted in 2012–14 in all states and union territories in India except the for the states of Bihar, Chhattisgarh, Jharkhand, Madhya Pradesh, Odisha, Rajasthan, Uttar Pradesh, Uttarakhand and Assam. States that were not covered in the DLHS household survey were instead covered through the government’s AHS, conducted in 2012–13 [26]. Together, the DLHS-4 and AHS provide coverage of districts across all Indian states in 2012–2014. NFHS-4 was conducted in 2015–16 [27] and provides data on household characteristics with district linkages. NFHS employed a multi-stage stratified sampling scheme and were designed to be representative at the state and national levels. Strata were defined by urban-rural setting and the primary sampling units (PSU) were villages in the rural stratum and wards in the urban stratum. NFHS provides data for all of the districts under study, with the advantage of having identical measures across all districts.

### Contextual indicators

The CICI framework was developed to provide guidance on the interacting dimensions of context, implementation, and settings that may impact the successful delivery of complex interventions [23]. Critically, the framework describes how upstream contextual factors beyond the organizational context may impact the implementation of a complex intervention with community-facing components. CICI considers 7 domains of context—geographical, epidemiological, socio-cultural, socioeconomic, ethical, legal, political. Here, we investigate the interplay among the CICI described political (healthcare infrastructure), epidemiological (measures of blood pressure, body mass index, older population), socioeconomic (aggregate community wealth), and geographical (region, urbanicity) domains. Although socio-cultural, ethical, and legal aspects warrant consideration, they are not easily quantifiable and are not included in the present study.

### Healthcare infrastructure

The CICI places healthcare infrastructure within the political domain because it is dependent on healthcare financing and regulations. As healthcare infrastructure is the sine qua non of medical management of blood pressure, this is the focal domain of this study. Healthcare system infrastructure indicators were developed based on a combination of the Indian Public Health Standards (2012 revision) [28]. We used the DLHS facility data to separately quantify the availability of diagnostics (blood pressure instrument), antihypertensive medication, potential staffing (medical officer, staff nurse, pharmacist, and community health worker), and specific infrastructure needed to support digital health initiatives (power supply, computer, internet connection). Indicators were contingent on the facility tier (DH, CHC, PHC, SCs). For example, at the district hospital level, a medical officer, public health nurse, and a pharmacist are designated as essential staff, whereas in health sub-centres (lowest level), only a female health worker is designated as essential. We only included personnel who, from our experience, are likely to be involved in hypertension care. The availability of indicators across tier of the healthcare system hierarchy is described in Supplemental Table 1.

### Epidemiologic, socioeconomic and geographical context

To assess the alignment of healthcare infrastructure with broader contextual characteristics, we also considered measures of the epidemiologic, socioeconomic, and geographical context. Such contextual indicators may have a bearing on allocation of health system resources. For example, health system infrastructure relevant to blood pressure may be directed to communities with greater epidemiologic burden of hypertension and its risk factors. Alternatively, it may be the case that wealthier communities may be the recipients of larger public investments in health.

Districts were characterized for epidemiologic and geographical context by aggregating household and individual data from the combined DLHS and AHS datasets. For epidemiologic indicators, we computed the mean systolic blood pressure and the mean body mass index by district. Geographical context was defined by region as well as the proportion of the population that resides in an urban area. Districts were classified as belonging to one of six regions—north (RJ, UT, HR, PB, HP, CH), west (MH, GA), south (TN, AP, AN, KL, KA, PY), central (CG, MP, UP), east (BR, JH, OR, WB) and northeast (AR, AS, MN, MZ, ML, NL, SK, TR)—according to the Indian Census. For socioeconomic context, we assigned each district the mean of measure of household wealth, a standardized asset-based measure of relative household wealth (mean = 0 and SD = 1) computed separately for urban and rural households. District-level wealth scores were derived from NFHS-4, for a measure of relative wealth comparable across all districts.

### Statistical analysis

Districts were the unit of analysis for this study. All statistical analyses were conducted in SAS v9.4 (SAS Institute; Cary, NC). Missing data in the facility assessments were assumed to indicate the absence of that the factor; for example, facilities lacking data on internet availability were assumed to have no internet.

District infrastructure indicators were first computed for all health facilities separately. Health facilities were scored 1 for the presence or 0 for the absence of each indicator available for its facility type. A composite variable was created for each facility and was coded as 1 if a facility reported having all required indicators (diagnostics, medication, potential staffing, and IT infrastructure). District-level infrastructure scores were computed as the mean of health facility composite binary indicators for all facilities within a district. The mean of the overall CHC facility composite score therefore reflects the proportion of CHCs in a particular district that met all of the infrastructure criteria listed above.

We conducted a descriptive analysis of all indicators and composite scores and estimated means and 95% confidence intervals (CIs) for the nation and by region. We computed the Spearman correlation among all of the contextual indicators, including the mean composite healthcare system infrastructure availability, at the district level. We further conducted logistic regression analysis to estimate the association between epidemiologic and geographical context (exposures) and the composite measure of health facility infrastructure to support digital health technology interventions (outcome) at the facility level. Models were implemented using generalized estimating equations to account for clustering of districts within states and were estimated separately for each facility tier (i.e., DH, CHC, PHC, and SC).

Finally, because the DLHS-4 was fielded in 2012, we conducted a sensitivity analysis to assess the correlation between total staffing of essential personnel at CHCs in 2012 and 2018 at the state level. Due to data limitations, only state level comparisons of staffing were possible and we focus on the CHC due to its emphasized role in NCD care.

## Results

**Fig. 1:**
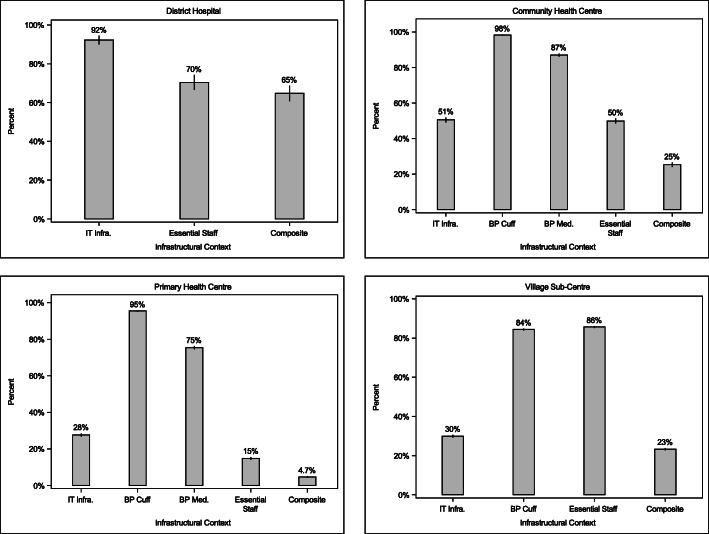
Infrastructural Context by Facility Tier across 512 districts in 29 states/UTs of India, 2012–14. Each bar is annotated with the point estimate of the national mean by facility tier, and the vertical lines on each bar marks the 95% confidence interval of the mean. A detailed description of indicators in each category by facility tier is shown in Supplemental Table 1

Data from a total of 18,334 SCs (lowest tier facility), 8526 PHCs, 4807 CHCs, and 548 DHs (highest tier facility) across 548 districts covering 29 states and union territories were analyzed. Figure 1 shows healthcare infrastructure by domain and facility tier. Among DHs, only 61% possessed all staff deemed minimally sufficient and essential. On the other hand, 92% of DHs had supporting IT infrastructure (internet availability was the only indicator surveyed at this level). Considering both essential staff and IT infrastructure, 57% of DHs were positioned to incorporate digital technologies for blood pressure management. Note that DHs were not surveyed for diagnostics and medications, ostensibly because these are presumed to be present within this facility tier. Among CHCs, 98% had BP instruments and 87% had antihypertensive medication. However, only 51% of CHCs possessed essential staff and IT infrastructure (regular power supply, facility computer, and working internet connection). Taking all of these elements together, 25% of CHCs were ready to undertake IT based interventions. PHCs showed a pattern similar to CHCs, with lower staffing and IT infrastructure. 96% of PHCs had BP instruments and 75% had some form of antihypertensive medication. However, only 15% had all essential staff positions filled, and only 28% had the IT infrastructure (regular power supply, facility computer, and working internet connection) expected at this level. Less than 5% of PHCs were ready for IT interventions for BP, the lowest readiness across facility type. Finally, SCs had BP instruments and essential staff (female health workers) in 94 and 88% of facilities, respectively. IT infrastructure (power supply) was relatively low at 30%. Overall, 23% of SCs were ready to undertake IT interventions for blood pressure.

**Fig. 2:**
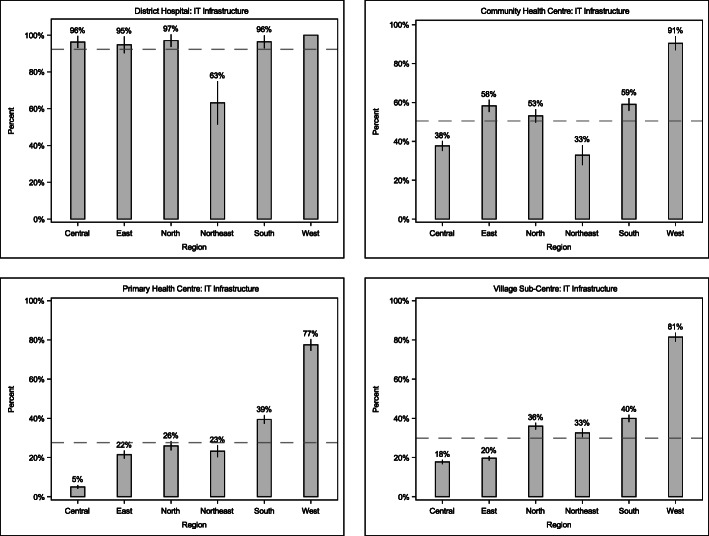
Infrastructure availability across healthcare facility tier and region in India, 2012–2014. Each bar is annotated with the point estimate of the national mean by region for each facility tier, and the vertical lines on each bar marks the 95% confidence interval of the mean. The horizontal dashed line shows the national mean for each facility tier. A detailed description of indicators in each category by facility tier is shown in Supplemental Table 1

Figure 2 shows the distribution of IT infrastructure availability, defined as a composite of all IT indicators available, by healthcare facility tier and region in 2012–2014. In general, we observed that IT infrastructure availability was highest for DHs and lower for CHCs, PHCs, and SCs. By region, districts in northeastern and central India tended towards having the least prepared health facilities with respect to IT infrastructure. The largest regional variability in infrastructure was observed at the levels of CHC and PHC, where IT infrastructure availability in the south and west statistically significantly exceeded the national mean while infrastructure for districts in central and northeast India was below the national mean.

Table 1 shows the correlation between the mean district composite infrastructure score and contextual indicators at the district level. DH infrastructure composite score was not correlated with the composite score of other facility tiers in the district, but was inversely correlated with average systolic blood pressure and positively correlated with the proportion of the population aged 60 years and older as well as the mean household wealth index. There were strong correlations among the CHC, PHC, and SC composite infrastructure scores, with correlations ranging from ranging from .29–.44. CHC, PHC, and SC composite infrastructure scores were also positively and significantly correlated with community-based mean systolic blood pressure and body mass index levels, the proportion of urban residents, and aggregate household wealth. The proportion of the population aged 60 years and older was not significantly associated with CHC or SC infrastructure.

**Table 1:** Correlations among health system infrastructure scores and contextual indicators

	*DH composite score*	*CHC composite score*	*PHC composite score*	*SC Composite score*	*Mean systolic blood pressure*	*Mean body mass index*	*Proportion of the population aged 60 y and older*	*Proportion of the population living in an urban setting*	*Mean household wealth index*
*DH composite score*	1.00000								
*CHC composite score*	0.05452 0.2381	1.00000							
*PHC composite score*	0.08205 0.0756	**0.29117 < .0001**	1.00000						
*SC Composite score*	−0.01702 0.7129	**0.44071 < .0001**	**0.29011 < .0001**	1.00000					
*Mean systolic blood pressure*	**−0.12143 0.0084**	**0.14008 0.0023**	**0.14351 0.0018**	**0.27024 < .0001**	1.00000				
*Mean body mass index*	−0.00495 0.9148	**0.31906 < .0001**	**0.45028 < .0001**	**0.43183 < .0001**	**0.43016 < .0001**	1.00000			
*Proportion of the population aged 60 y and older*	**0.09556 0.0384**	0.04012 0.3854	**0.13008 0.0047**	0.07249 0.1166	**0.18510 < .0001**	**0.15481 0.0008**	1.00000		
*Proportion of the population living in an urban setting*	−0.02449 0.5963	**0.20720 < .0001**	**0.22322 < .0001**	**0.18291 < .0001**	**0.17995 < .0001**	**0.39681 < .0001**	0.02546 0.5819	1.00000	
*Mean household wealth index*	**0.10203 0.0270**	**0.32090 < .0001**	**0.26117 < .0001**	**0.29096 < .0001**	**0.29572 < .0001**	**0.41076 < .0001**	**0.09506 0.0394**	**0.38796 < .0001**	1.00000

*Notes*: Correlations are shown among 470 districts with complete data on all indicators. Variables summarize each indicator at the district level. The Spearman correlation is shown as the first row of each cell. The second row shows the *p*-value for the correlation. Bold values indicate *p* < .05

*CHC* Community health centre, *DH* District hospital, *PHC* Primary health centre, *SC* sub-centre

Table 2 shows the association of epidemiologic, socioeconomic, and geographic contextual indicators with district-level composite infrastructure measures from adjusted logistic regression models accounting for all other context characteristics. Among DHs, the population aged over 60 y was significantly and positively associated with DH infrastructure (adjusted OR [aOR] = 1.74; 95%CI: 1.05–2.90). Among CHCs, location in a Western state, mean BMI, mean SBP, and percent of the population that was urban were all positively and statistically significantly related to the composite infrastructure score, suggesting that the availability of CHC infrastructure was aligned with populations with higher levels of blood pressure at the time of survey. Similar associations were seen in adjusted models among PHCs, but household wealth was the only community factor statistically significant in the adjusted model of PHC infrastructure (aOR = 5.89; 1.41–24.59). PHCs in the southern and western regions were much more likely than those in the central region to possess all the infrastructure elements needed for digital health technologies in BP management. The regional pattern was also apparent for SCs, which were more likely to have better infrastructure in the west. Population BMI and urbanicity, but not wealth, were associated with more favorable SC infrastructure.

**Table 2:** Associations (odds ratios and 95% CI) of Geographic and Epidemiologic Context with Composite Health Facility Infrastructure to Support Digital Health Technology Interventions

	District Hospital		Community Health Centre		Primary Health Centre		Sub-Centre	
	Unadjusted	Adjusted	Unadjusted	Adjusted	Unadjusted	Adjusted	Unadjusted	Adjusted
Region (ref = Center)								
East	0.79 (0.28–2.19)	0.97 (0.34–2.73)	1.96 (0.36–10.78)	1.73 (0.41–7.34)	0.78 (0.09–6.49)	1.72 (0.13–22.36)	1.12 (0.22–5.70)	1.62 (0.38–6.96)
North	1.21 (0.40–3.66)	1.10 (0.36–3.39)	2.49 (0.84–7.41)	1.74 (0.59–5.16)	5.95 (0.71–50.15)	1.15 (0.12–11.26)	2.31 (0.56–9.54)	0.86 (0.22–3.32)
Northeast	0.40 (0.11–1.53)	0.56 (0.15–2.09)	1.06 (0.33–3.42)	0.84 (0.24–2.89)	**14.00 (1.64–119.4)**	**13.93 (1.71–113.4)**	1.64 (0.45–5.95)	1.33 (0.39–4.55)
South	1.29 (0.35–4.77)	1.70 (0.36–8.11)	2.86 (0.69–11.83)	1.43 (0.33–6.21)	**20.99 (2.90–152.1)**	**7.10 (1.03–48.78)**	**1.49 (0.38–5.86)**	**1.74 (1.05–2.90)**
West	1.61 (0.61–4.26)	1.39 (0.41–4.69)	**5.96 (2.04–17.46)**	**3.63 (1.31–10.06)**	**11.69 (2.09–65.42)**	**3.91 (0.60–25.65)**	**16.97 (5.04–57.09)**	**9.73 (2.64–35.93)**
Population over age 60^a^	**1.64 (1.02–2.63)**	**1.74 (1.05–2.90)**	1.13 (0.62–2.07)	0.83 (0.51–1.36)	1.83 (0.82–4.06)	1.27 (0.67–2.39)	**1.10 (1.03–1.17)**	1.05 (0.99–1.13)
Mean body mass index	1.00 (0.84–1.18)	0.94 (0.77–1.15)	**1.36 (1.11–1.67)**	**1.21 (1.05–1.39)**	**1.81 (1.37–2.39)**	1.35 (1.14–1.59)	**1.43 (1.12–1.83)**	**1.23 (1.01–1.52)**
Mean systolic blood pressure	0.95 (0.90–1.01)	**0.92 (0.87–0.97)**	**1.08 (1.04–1.11)**	1.03 (0.99–1.07)	**1.16 (1.04–1.29)**	1.03 (0.94–1.13)	**1.02 (1.00–1.03)**	1.00 (0.99–1.01)
% Urban	0.99 (0.98–1.01)	**0.99 (0.97–1.00)**	**1.02 (1.01–1.03)**	**1.01 (1.00–1.02)**	**1.02 (1.00–1.04)**	1.00 (0.99–1.01)	**2.57 (1.47–4.51)**	**2.02 (1.24–3.30)**
Household wealth	1.51 (0.97–2.35)	**2.07 (1.29–3.33)**	1.84 (0.96–3.54)	1.06 (0.65–1.72)	**4.35 (1.23–15.31)**	**5.89 (1.41–24.59)**	1.23 (0.63–2.39)	0.78 (0.53–1.16)

The table shows odds ratios and 95% CI from models at the facility level adjusted for clustering within states. Adjusted models included all variables in the leftmost column simultaneously. Sample sizes are as follows: 548 district hospitals; 4807 community health centres; 8526 primary health centres, and 18,334 sub-centres

^a^ This variable was dichotomized so that the association compares districts with over 16.9% of the population over the age of 60 (the 75th percentile of this variable) to districts with lesser proportions of adults aged 60 years and older. This variable was dichotomized for the modeling to provide stability to the standard errors

Comparing the total staffing for essential personnel relevant to hypertension management in CHCs, we observed statistically significant and positive correlations at the state level among the number of staff nurses (*r* = .41; *p* = .035), medical officers (*r* = .68; <.001), and pharmacists (*r* = .846; *p* < .001) in position between 2012 and 2018.

## Discussion

This study used a contextual framework to evaluate potential gaps in infrastructure needed to implement digital technologies for hypertension screening and management within the government health care system. We identified and quantified several constraints to implementing digital health interventions for blood pressure management within the government sector in India in 2012–2014. Our examination revealed that shortfalls in essential staff may be a larger barrier to these programs than the availability of IT-specific infrastructure. We also observed that gaps were generally larger for lower tier facilities and for facilities in northeastern and central India, and these gaps substantially varied by region of the nation. As a potential area of strength for the health system, we noted that the availability of all healthcare system infrastructural elements (diagnostics, medications, staff, and IT infrastructure) tended to be aligned with the location of higher need for blood pressure management: districts with higher average SBP and BMI were more likely to meet the composite infrastructure criteria. These data provide early benchmarks for state planning and allocation for resources at the district level.

Since the DLHS-4, there has been major public investment in using digital technologies to strengthen care for hypertension as well as other priority NCD. To mobilize awareness of priority NCDs among communities for whom there is low culture of engagement with formal preventative healthcare, the Ministry of Health & Family Welfare, Government of India, published operational guidelines in 2017 to promote universal community screening of hypertension, diabetes, and three common cancers among all adults over the age of 30 years. To identify previously undiagnosed cases of hypertension and identify high risk adults, lay community health workers, Accredited Social Health Activists (ASHAs), are incentivized by the government to support auxiliary nurse midwives and other formal healthcare providers through door-to-door screening of adults.

From the ASHA to the staff nurse to the medical officer, digital applications to assist healthcare personnel to record and track patient outcomes and provide referral and management prompts are now available. These digital health tools require trained users, along with appropriate blood pressure screening devices, anti-hypertensive medications, and IT-supportive infrastructure such as power supply and internet. Our findings indicate that personnel shortages loomed larger than technological barriers prior to the take off of these programs. It remains to be seen how either human or technological resources have been impacted by major recent shifts, like the COVID-19 pandemic.

### Strengths and limitations

A major strength of our study was the use of the CICI framework, which provided a conceptual lens to inform our analysis. Furthermore, we employed multiple datasets to address the framework.

The study also had several limitations. While we used the most recent data available, the facility assessments occurred in 2012–2014, prior to the initiation of many of the digital health initiatives in play today. The finding that human resources may be a larger limitation than the lack of IT capacity or other supplies may no longer hold true due to subsequent investments in this area. Epidemiologic and socioeconomic indicators came from datasets that surveyed the Indian population during (AHS: 2012–13) and after (NFHS: 2015–16) the data collection period for the DLHS infrastructure data (2012–2014). In particular, the socioeconomic context captured in NFHS may have improved in the year since the DLHS was completed. There are also major limitations to the generalizability of our findings. First, our study is restricted to states and union territories with facilities data available and to the government healthcare sector. Specifically, Gujarat, Jammu and Kashmir, Dadra and Nagar Haveli, Daman and Diu, Delhi and Lakshadweep—roughly accounting for 8% of the Indian population according to the 2011 Census—were excluded from this study. The excluded areas are socioeconomically and demographically diverse, and there are no consistent differences between excluded and included areas. Secondly, these findings apply to public sector healthcare. Many Indians report not utilizing government healthcare due to perceptions of including poor quality of care, doctor unavailability, drug unavailability, absence of healthcare personnel and lack of adequate infrastructure [29]. Analyses such as ours provide quantification of specific gaps within the government sector but do not address capacities (or deficits) in the private sector.

## Conclusions

As India and other nations deploy plans to incorporate digital health technologies, our study provides a model for assessment of pre-implementation barriers that can be used to advocate for health systems strengthening. Such empirical investigation may challenge pre-conceived notions regarding the gaps in implementing novel intervention delivery strategies, such as digital technologies. A structural perspective on the environment around incorporating novel technologies into the delivery of care for hypertension and other chronic disease may accelerate our ability to identify and address gaps in the healthcare system and beyond. Future studies may investigate the presence and absence of necessary infrastructure using contemporary data. Furthermore, there is an opportunity to further evaluate the CICI for use in LMICs, as has been done for other implementation science frameworks [30].

## Supplementary Information

**Additional file 1: Supplemental Table 1.** National distribution of each available indicator by domain and facility type.

## Data Availability

The data used in this study are publicly available. Both the District Level Health Survey and the National Family Health Survey data are available from the International Institute of Population Sciences, India (http://www.iipsindia.org).

## Abbreviations

AHS: Annual Health Survey
ASHA: Accredited Social Health Activist
BMI: Body Mass Index
BP: Blood Pressure
CHC: Community Health Centre
CI: Confidence Interval
CICI: Context and Implementation of Complex Interventions
DH: District Hospital
DLHS: District Level Household and Facility Survey
IPHS: Indian Public Health Standards
IT: Information Technology
LMIC: Lower- and Middle-Income Country
NCD: Non-Communicable Diseases
NFHS: National Family Health Survey
PHC: Primary Health Centre
PSU: Primary Sampling Unit
AN: Andaman and Nicobar Islands
AP: Andhra Pradesh
AR: Arunachal Pradesh
AS: Assam
BR: Bihar
CG: Chhattisgarh
CH: Chandigarh
GA: Goa
HP: Himachal Pradesh
HR: Haryana
JH: Jharkhand
KA: Karnataka
KL: Kerala
MH: Maharashtra
ML: Meghalaya
MN: Manipur
MP: Madhya Pradesh
MZ: Mizoram
NL: Nagaland
OR: Odisha
PB: Punjab
PY: Puducherry
RJ: Rajasthan
SK: Sikkim
TN: Tamil Nadu
TR: Tripura
UP: Uttar Pradesh
UT: Uttarakhand
WB: West Bengal
