# Supplementary material for: A model for national assessment of barriers for implementing digital technology interventions to improve hypertension management in the public health care system in India
Source: BMC Health Serv Res. 2021 Oct 15;21:1101. doi: 10.1186/s12913-021-06999-9 (PMC8517936; doi:10.1186/s12913-021-06999-9)
Supplement: Supplementary file 1 — Additional file 1: Supplemental Table 1. National distribution of each available indicator by domain and facility type. [file 12913_2021_6999_MOESM1_ESM.docx]

# SUPPLEMENTAL TABLES AND FIGURES

## Supplemental Table 1. National distribution of each available indicator by domain and facility type

| Facility tier | Domain | variable | Prevalence (95% CI) across districts |
| --- | --- | --- | --- |
| 1. District Hospital (apex government facility at the district level) | IT infrastructure | Internet Available* | 92.3(90.1-94.6) |
|  | Essential staff | Medical Officer | 86.5 (83.6-89.4) |
|  | Essential staff | Pharmacist | 91.6 (89.3-93.9) |
|  | Essential staff | Staff Nurse | 89.4 (86.8-92) |
|  | Essential staff | DH: Essential Staff | 70.4 (66.6-74.3) |
|  | Summary score | DH Composite | 64.8 (60.8-68.8) |
| 2. Community Health Centre (mid-tier facility with primary responsibility for NCD care) | Medication | Antihypertensive | 87 (86-87.9) |
|  | Diagnostics | BP instrument | 98.3 (98-98.7) |
|  | Essential staff | Medical Officer | 82.7 (81.7-83.8) |
|  | Essential staff | Pharmacist | 80.2 (79-81.3) |
|  | Essential staff | Staff Nurse | 72.7 (71.5-74) |
|  | Essential staff | CHC: Essential Staff | 49.9 (48.5-51.3) |
|  | IT infrastructure | Computer Available | 91 (90.2-91.8) |
|  | IT infrastructure | Internet Available | 84.3 (83.3-85.4) |
|  | IT infrastructure | Regular Power Supply | 57.8 (56.4-59.2) |
|  | IT infrastructure | IT Infrastructure composite | 50.5 (49.1-51.9) |
|  | Summary score | CHC: Composite | 25.3 (24.1-26.5) |
| 3. Primary Health Centre (mid-tier facility) | Medication | Antihypertensive medication | 75.4 (74.5-76.4) |
|  | Diagnostics | BP instrument | 95.5 (95-95.9) |
|  | Essential staff | Medical Officer | 69.5 (68.5-70.5) |
|  | Essential staff | Pharmacist | 60.7 (59.6-61.7) |
|  | Essential staff | Staff Nurse | 31 (30.1-32) |
|  | Essential staff | PHC: Essential Staff | 14.8 (14.1-15.6) |
|  | IT infrastructure | Computer Available | 56.2 (55.1-57.2) |
|  | IT infrastructure | Internet Available | 46.6 (45.6-47.7) |
|  | IT infrastructure | Regular Power Supply | 49.6 (48.6-50.7) |
|  | IT infrastructure | IT Infrastructure composite | 27.6 (26.7-28.6) |
|  | Summary score | PHC: Composite | 4.7 (4.2-5.1) |
| 4. Village Sub-Centre (lowest tier facility at the village-level | Diagnostics | BP instrument | 84.4 (83.9-84.9) |
|  | IT infrastructure | Regular Power Supply* | 29.9 (29.3-30.6) |
|  | Essential staff | Female Worker | 85.7 (85.2-86.2) |
|  | Summary score | SC: Composite | 23.3 (22.7-23.9) |

*The only measured indicator for that domain at that tier.

All data are from the DLHS-4 (2012-1014).

Sub-Centres by design do not have medications on site, and district hospitals were not surveyed on medication or diagnostics.
